# The impact on poisonings of up‐scheduling of modified release paracetamol to Schedule 3 (pharmacist only medicine)

**DOI:** 10.5694/mja2.51888

**Published:** 2023-03-20

**Authors:** Rose Cairns, Firouzeh Noghrehchi, Nicholas A Buckley

**Affiliations:** ^1^ The University of Sydney Sydney NSW; ^2^ NSW Poisons Information Centre Children's Hospital at Westmead Sydney NSW

**Keywords:** Pharmacoepidemiology, Policy, drugs and alcohol, Analgesics, Self‐injurious behavior

In Australia, paracetamol is the agent most frequently implicated in drug overdoses, and their frequency is increasing, particularly in young people.^1^ Paracetamol overdose causes significant morbidity despite treatment, and is the leading cause of acute liver failure in Western countries.^2^ Modified release (MR) paracetamol overdose is associated with a higher rate of liver injury than immediate release paracetamol.^3^ The sole therapeutic benefit of MR paracetamol is its more convenient dosage regimen (three rather than four times a day).^4^

In response to the rising numbers of overdoses, the Therapeutic Goods Administration (TGA) up‐scheduled MR paracetamol, from Schedule 2 to Schedule 3, in June 2020.^5^ A pharmacist must be involved in sales of Schedule 3 medicines, which must be stored behind the pharmacy counter. We evaluated whether re‐scheduling was associated with changes in the numbers of overdoses with MR paracetamol, immediate release paracetamol, and other over‐the‐counter analgesics reported to the New South Wales Poisons Information Centre (NSWPIC) to the end of August 2022. The study was approved by the Sydney Children's Hospitals Network Human Research Ethics Committee (2021/ETH00165).

We assessed changes in monthly overdose numbers using interrupted time series analysis. We also examined the impact of coronavirus disease 2019 (COVID‐19)‐related restrictions, and that of new paracetamol overdose treatment guidelines that could have increased referrals to NSWPIC (published online in December 2019^3^), using changepoint analysis (details: Supporting Information).

A total of 1715 exposures to MR paracetamol were reported to NSWPIC during 1 February 2017 – 31 August 2022, 914 during the 40 months preceding re‐scheduling (mean, 23 calls per month) and 801 during the 27 months after re‐scheduling (mean, 30 calls per month); 1081 reports concerned adults (63%), 215 children aged 5–14 years (12%), and 412 adolescents (24%) (Supporting Information, table 1).

Interrupted time series analysis identified no significant level or slope change in MR paracetamol overdose number after re‐scheduling, whereas the number of reports regarding other over‐the‐counter analgesics increased significantly (Box 1, Box 2). Changepoint analysis identified an increase in MR paracetamol overdoses in December 2019; that is, prior to re‐scheduling. The increase in immediate release paracetamol overdoses (March 2020) preceded re‐scheduling, and coincided with a COVID‐19 lockdown in Sydney. Increased numbers of overdoses with ibuprofen (May 2020) and other over‐the‐counter analgesics (June 2020) may have been related to COVID‐19 restrictions or to the re‐scheduling of MR paracetamol (Supporting Information, table 2). The median MR paracetamol dose involved in overdoses declined slightly, from 13.3 g (interquartile range [IQR], 7.3–23.9 g) before to 11.8 g (IQR, 6.7–22.3 g) after re‐scheduling (Supporting Information, figure).

Box 1 Intentional poisonings with over‐the‐counter analgesics reported to the New South Wales Poisons Information Centre, 1 February 2017 – 31 August 2022: interrupted time series analysis*

**Figure:**
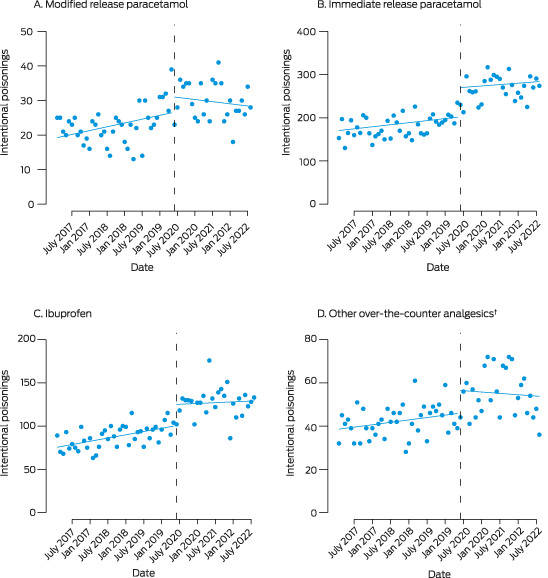
* Reported (points) and predicted (lines) poisonings; dashed vertical line: up‐scheduling of modified release paracetamol on 1 June 2020. † Naproxen, diclofenac, mefenamic acid, aspirin, paracetamol/ibuprofen combinations.

Box 2 **Intentional poisonings with over‐the‐counter analgesics: estimates derived from interrupted time series analysis**

**Table:** 

Drug	Monthly slope before schedule change* (95% CI)	Level change after schedule change* (95% CI)	Change in slope after schedule change* (95% CI)
Modified release paracetamol	0.18 (0.05 to 0.32)	4.67 (–0.28 to 9.6)	–0.29 (–0.57 to 0.00)
Immediate release paracetamol	0.79 (0.22 to 1.37)	66.1 (49.2 to 87.0)	–0.26 (–1.38 to 0.87)
Ibuprofen	0.63 (0.27 to 0.98)	25.0 (11.9 to 38.1)	–0.47 (–1.21 to 0.26)
Other over‐the‐counter analgesics^†^	0.19 (–0.04 to 0.42)	10.8 (2.4 to 19.2)	–0.29 (–0.76 to 0.18)

CI = confidence interval.

* 1 June 2020.

† Naproxen, diclofenac, mefenamic acid, aspirin, paracetamol/ibuprofen combinations.

We found that poisonings with MR paracetamol did not significantly decline after its re‐scheduling as a Schedule 3 medicine; the number was increasing prior to re‐scheduling, and continued to do so subsequently. The increase in overdoses with ibuprofen and other over‐the‐counter analgesics may indicate that people were switching analgesics; as MR paracetamol is more toxic than non‐steroidal anti‐inflammatory drugs, this would be consistent with harm reduction.

Further MR paracetamol restrictions are being considered by the TGA to reduce the risks of overdose. The European Medicines Agency has already banned MR paracetamol.^6^ The TGA scheduling delegate has rejected advice from the Independent Expert Report^1^ and the Royal Australian College of General Practitioners^7^ to re‐schedule MR paracetamol to Schedule 4, citing insufficient time since the previous re‐scheduling.^5^ Other studies have found that up‐scheduling of drugs has an immediate impact on poisoning rates.^8^ The TGA scheduling delegate also noted that most people with osteoarthritis (presumed MR paracetamol users) are over 55 years of age, and therefore not in the main risk group for overdose.^9^ We found, however, that more than one‐third of MR paracetamol overdoses were in children or adolescents, suggesting substantial casual use of a medicine intended for treating chronic pain. Further up‐scheduling would probably reduce casual use and consequently the number of overdoses.

## Open access

Open access publishing facilitated by The University of Sydney, as part of the Wiley – The University of Sydney agreement via the Council of Australian University Librarians.

## Competing interests

Rose Cairns holds an untied educational grant from Reckitt; this funder had no role in this study.

## Supporting information


**Data S1** Supporting Information
