# Supplementary material for: The impact on poisonings of up‐scheduling of modified release paracetamol to Schedule 3 (pharmacist only medicine)
Source: Med J Aust. 2023 Mar 20;218(9):412–4. doi: 10.5694/mja2.51888 (PMC10952151; doi:10.5694/mja2.51888)
Supplement: Supplementary file 1 — Data S1 Supporting Information [file MJA2-218-412-s001.pdf]

The Medical Journal of Australia

## Supporting Information

## Supplementary methods and results

**This appendix was part of the submitted manuscript and has been peer reviewed.**
**It is posted as supplied by the authors.**

Appendix to: Cairns R, Noghrehchi F, Buckley NA. The impact on poisonings of up-scheduling of modified release paracetamol to Schedule 3 (pharmacist only medicine). *Med J Aust* 2023; doi: 10.5694/mja2.51888.

## Supplementary methods

*Data source and data extraction*

New South Wales Poisons Information Centre (NSWPIC) is the largest Poisons Information Centre in Australia, receiving approximately 50% of the national 220,000 annual poisoning calls from health care professionals and members of the public[1]. Approximately 65% of NSWPIC calls originate from NSW. We analysed data from the NSWPIC database related to poisonings with over-the-counter analgesics. All single-ingredient paracetamol poisonings (MR and immediate release) are coded under the single "paracetamol" substance code; to identify MR paracetamol poisonings we conducted a free text search of the product and dose fields for *665*, *MR*, *SR*, *CR*, *XR*, *osteo*, *sustain*, *modified*. Records returned were reviewed manually and re-coded. We also extracted calls for other analgesics available without prescription, including ibuprofen, diclofenac, mefenamic acid, naproxen, aspirin, and paracetamol/ibuprofen combinations. Intentional poisoning calls were defined as calls coded as "deliberate self-poisoning" or "intentional: other". Only exposure calls were counted (that is, re-calls and subsequent calls about the same event were excluded).

*Interrupted time series analysis*

We used interrupted time series analysis to assess changes in monthly poisoning calls following the up-scheduling of MR paracetamol from Schedule 2 to Schedule 3 in June 2020. Interrupted time series analysis is one of the strongest quasi-experimental designs for evaluating the effectiveness of population-level health interventions commenced at a defined time point.[2] Our outcome variable, the number of poisoning calls, follows a Poisson distribution. However, when the expected count number is large and the distribution is not bounded by zero, a Poisson distribution can be approximated by a normal distribution. We therefore applied segmented linear regression, which assumes a continuous outcome, to model the intervention.

We used a combination of the Durbin–Watson test, the Ljung–Box test for white noise, and the autocorrelation function and partial autocorrelation function plots to test for the presence of auto-correlation in our models. If any of these tests indicated auto-correlation, we included autoregressive terms in the base model to control for it. We estimated the models using the *arima* function in the stats R-package. We specified the model order as (p,0,0), with p representing the autoregressive order. We investigated for seasonality by including dummy variables for the months and Fourier terms in our models. We checked the normality assumption and the homoscedasticity assumption of residuals underlying linear regression by inspecting the residual plots against time and against the fitted values, and the normal quantile–quantile plot of residuals. All the analyses were performed in R 4.0.1.

*Structural changepoint analysis*

Because the up-scheduling of MR paracetamol was implemented shortly after the start of the COVID-19 pandemic and related lockdowns started in NSW, we conducted structural changepoint analysis to identify whether changes in the monthly poisoning call numbers may have preceded up-scheduling, using multiple structural changepoint modelling[3] and the breakpoints function in the *strucchange* R-package. We fitted time series models adjusted for auto-correlation as necessary and seasonality according to the two approaches described above, and chose the most parsimonious model based on Akaike information criterion (AIC) and after checking other model assumptions (normality and homoscedasticity). We applied multiple structural change modelling to the model chosen by AIC and computed the optimal break points and their 95% confidence intervals (CIs). The changepoint analysis estimates the breakpoint; its variance, a measure of the uncertainty in the estimate, provides the 95% CI. The null hypothesis was no change or break in the series. We allowed for the number of breakpoints to be more than one. Finally, we performed interrupted time series analysis as described above with the identified optimal breakpoint as the intervention date. For each outcome, only one breakpoint was identified.

## Supplementary results

**Table 1. Demographic characteristics of people involved in intentional modified release paracetamol exposures reported to the New South Wales Poisons Information Centre**

| | **Pre-intervention**<br>1 Feb 2017 – 31 May 2020 | **Post-intervention**<br>1 June 2020 – 31 Aug 2022 |
|---|---|---|
| **Number of reports** | 914 | 801 |
| **Age (years), median (IQR)*** | 24 (16-47) | 25 (15-50) |
| Children (5–14 years) | 90 (9.8%) | 125 (15.6%) |
| Adolescents (15–19 years) | 231 (25.3%) | 181 (22.6%) |
| Adults (20–74 years) | 546 (59.7%) | 456 (56.9%) |
| Older adults(75 or more years) | 43 (4.7%) | 36 (4.5%) |
| Unknown | 4 (0.4%) | 3 (0.4%) |
| **Sex** | | |
| Females | 655 (71.7%) | 576 (71.9%) |
| Males | 255 (27.9%) | 221 (27.6%) |
| Unknown | 4 (0.4%) | 4 (0.5%) |

IQR = interquartile range.

*Exact age missing for 138 pre-intervention and 95 post-intervention calls.

**Table 2. Intentional poisonings with over-the-counter analgesics: autoregressive models, before and after optimal breakpoint estimated by structural change point analysis**

| **Drug** | **Breakpoint*** **(95% CI)** | **Auto-regressive order†** | **Monthly slope before break (95% CI)** | **Level change after break (95% CI)** | **Change in slope after break (95% CI)** |
|---|---|---|---|---|---|
| Modified release paracetamol | Dec 2019<br>(Aug 2019 – Mar 2020) | AR(1) | –0.02<br>(–0.18 to 0.14) | 10.2<br>(5.8–14.6) | –0.06<br>(–0.29 to 0.16) |
| Immediate release paracetamol | Mar 2020<br>(Jan 2020 – May 2020) | AR(2) | 0.73<br>(–0.03 to 1.49) | 46.3<br>(21.8–70.7) | 0.92<br>(–0.38 to 2.21) |
| Ibuprofen | May 2020<br>(Nov 2019 – Jun 2020) | AR(2) | 0.61<br>(0.24–0.99) | 22.9<br>(9.7–36.1) | –0.30<br>(–1.02 to 0.42) |
| Other over-the-counter analgesics‡ | Jun 2020<br>(Dec 2019 – Oct 2020) | AR(1) | 0.19<br>(–0.04 to 0.42) | 10.8<br>(2.4–19.2) | –0.29<br>(–0.76 to 0.18) |

*CI = confidence interval.*

* A breakpoint is the first month after the structural change detected by the model.

† An autoregressive model, AR(p), regresses the outcome on its own past values and includes p number of lags.

‡ Naproxen, diclofenac, mefenamic acid, aspirin, paracetamol/ibuprofen combinations.

**Figure. Violin plot showing dose taken in modified release paracetamol overdoses, before and after re-scheduling. Dashed line shows median and dotted lines show upper and lower quartiles.**

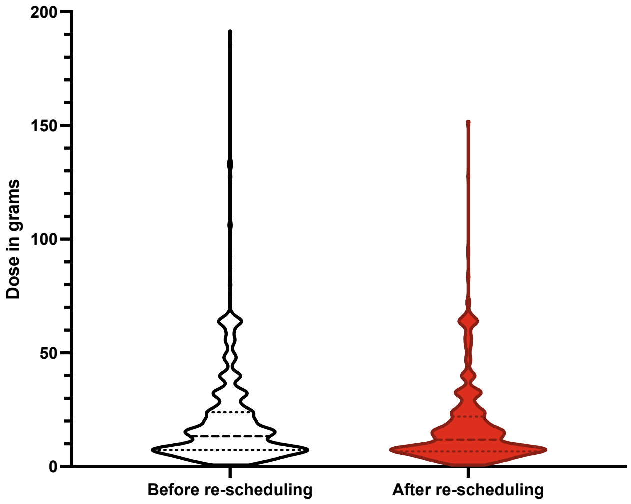


There was a decrease in proportion of poisonings which involved MR paracetamol following re-scheduling: MR paracetamol accounted for 6.9% of OTC analgesic poisonings pre-intervention, and 6.2% post-intervention (p= 0.02, Pearson's chi-squared). There was a slight reduction in median dose taken associated with the re-scheduling, from 13.3 g (IQR 7.3, 23.9 g) to 11.8 g (IQR 6.7, 22.3 g), p=0.008 (Mann-Whitney) (figure 1).

## Supplementary references

1. Poisons Information Centres [published 2020 Aug]. In: Therapeutic Guidelines. Melbourne: Therapeutic Guidelines Limited; https://www.tg.org.au [accessed February 2023].
2. Cook TD, Campbell DT, Day A. Quasi-experimentation: Design & analysis issues for field settings. Boston: Houghton Mifflin; 1979 Jul.
3. Bai J, Perron P. Computation and analysis of multiple structural change models. *J Appl Econ.* 2003; 18: 1-22.
